# Investigating the anticancer efficacy of biogenic synthesized MgONPs: An *in vitro* analysis

**DOI:** 10.3389/fchem.2022.970193

**Published:** 2022-09-15

**Authors:** Shams Tabrez, Azhar U. Khan, Mehboob Hoque, Mohd Suhail, Mohammad Imran Khan, Torki A. Zughaibi

**Affiliations:** ^1^ King Fahd Medical Research Center, King Abdulaziz University, Jeddah, Saudi Arabia; ^2^ Department of Medical Laboratory Sciences, Faculty of Applied Medical Sciences, King Abdulaziz University, Jeddah, Saudi Arabia; ^3^ Department of Chemistry, School of Life and Basic Sciences, SIILAS CAMPUS, Jaipur National University, Jaipur, India; ^4^ Applied Bio-Chemistry Lab, Department of Biological Sciences, Aliah University, Kolkata, India; ^5^ Department of Biochemistry, Faculty of Science, King Abdulaziz University, Jeddah, Saudi Arabia

**Keywords:** anticancer, biogenic, cytotoxicity, electron microscopy, MgONPs

## Abstract

The biogenic approach of synthesizing metal nanoparticles is an exciting and interesting research area with a wide range of applications. The present study reports a simple, convenient, low-cost method for synthesizing magnesium oxide nanoparticles (MgONPs) from pumpkin seed extracts and their anticancer efficacy against ovarian teratocarcinoma cell line (PA-1). The characteristic features of biogenic MgONPs were assessed by UV–visible spectrophotometry (UV–vis), X-ray powder diffraction (XRD), scanning electron microscopy (SEM), and transmission electron microscopy (TEM). The formation of spherical NPs with an average size of 100 nm was observed by scanning electron microscopy (SEM) and transmission electron microscopy (TEM). Moreover, MgONPs exhibit considerable cytotoxicity with an IC_50_ dose of 12.5 μg/ml. A dose-dependent rise in the induction of apoptosis, ROS formation, and inhibition in the migration of PA-1 cells was observed up to 15 μg/ml concentration, reflecting their significant anticancer potential against ovarian teratocarcinoma cell line. However, additional work, especially in different *in vitro* and *in vivo* models, is recommended to find out their real potential before this environment-friendly and cost-effective nanoformulation could be exploited for the benefit of humankind.

## Introduction

Cancer is a multifaceted disease caused by a complex interplay of genetic and environmental factors, highly variable in presentation, development, and outcome (Somarelli, 2021). It continues to be a global health concern with a significant number of mortalities (Siegel et al., 2022). Conventional anticancer therapeutics have their limitations, which include insufficient bioavailability, inadequate pharmacological effect, and irreversible and undesirable injury to healthy tissues and cells (Buttacavoli et al., 2018; Tabrez et al., 2020).

Because of their ability to overcome the above-mentioned limitations, nanotechnology has rapidly gained importance in the biomedical field, ultimately helping to manage/control life-threatening diseases (Kemp and Kwon, 2021; Tabrez et al., 2022a). It has applications in different fields, such as electronics, textile, and healthcare, that include targeted drug delivery, diagnosis, and biosensing treatment for the welfare of humanity (Akhtar et al., 2013; Khan et al., 2018; Alserihi et al., 2022). The nanotechnology-based formulation has shown improved stability and biocompatibility, enhanced permeability and retention effect, and precise targeting to cancer sites (Kim et al., 2021; Sharifi-Rad et al., 2021). Among the various nanoformulations, the use of metal oxide nanoparticles in preventive medicine has risen due to their unique physicochemical characteristics, such as high surface/volume ratio, broad optical properties, ease of synthesis, surface functionalization, cost effectiveness, and environment-friendly nature (Tabrez et al., 2022b; Zughaibi et al., 2022).

Pumpkins are a gourd vine squeeze of the genus *Cucurbita* and family Cucurbitaceae and are grown widely around the world as a vegetable. Pumpkin seeds are small, oval, and have high nutritional values. Out of the total 27 species, three pumpkin species, namely, *Cucurbita maxima*, *Cucurbita pepo*, and *Cucurbita moschata*, are cultivated globally for (Phillips et al., 2005; Patel, 2013). The flesh and seeds of pumpkin contain a high amount of proteins, antioxidants (carotenoids and tocopherols), minerals, fat, and calories (Stevenson et al., 2007; Tabrez et al., 2022b; Zughaibi et al., 2022). Pumpkin seeds are also a dietary source of polyunsaturated fatty acids and phytosterols (e.g., β-sitosterols) and a good source of zinc (Ryan et al., 2007; Sabudak, 2007). The scientific literature indicates a great therapeutic potential for pumpkin seeds which have also been utilized as an alternative medicine (Batool et al., 2022). They are cheaper and used in many food products to increase their nutritional value. The health benefits associated with pumpkin seeds are control of blood glucose and cholesterol, improving immunity and liver functioning, solving gall bladder disabilities, maintaining prostate gland health, curing depression and inflammation, and cancer management (Yadav et al., 2010; Syed et al., 2019; Salehi et al., 2021).

Nanotechnology and magnesium oxide have been considered a valuable combination for cancer therapy. The experimental and epidemiological evidence suggested multiple roles of magnesium compounds in cellular metabolism and maintaining genetic stability, regulation of cell proliferation, and protection against insulin resistance, oxidative stress, and systemic inflammation (Liu and Dudley, 2020; Fiorentini et al., 2021).

Recent studies have reported different magnesium oxide nanoparticle (MgONP) biosynthesis methods owing to their catalytic characteristics, high thermal conductivity, and fire-resistant potential (Sharma et al., 2020; Hassan et al., 2021). Several studies have reported the synthesis of MgONPs from various plant parts and fungi (Amina et al., 2020; Essien et al., 2020; Hassan et al., 2021; Saied et al., 2021). A general acceptance of green-synthesized plant-based nanoparticles in the therapeutic regimen facilitated anticancer efficacy of different bioactive compounds. This has a convenient synthesis protocol with a limited use of toxic reagents and byproducts, along with minimum invasiveness and maximum biocompatibility (Bhardwaj et al., 2020). MgONPs are potentially utilized in interdisciplinary domains such as biodiesel production, thermal energy, chemical healing, and high-temperature thermochemical storage (Sharma et al., 2020; Dabhane et al., 2021). Biologically, MgONPs have been reported as non-toxic microbial inhibitors and a biocompatible material used in scaffolding for tissue engineering, dental cement, and cryosurgery of cancer cells (Di et al., 2012; Sharma et al., 2020). The scientific reports on ecofriendly and biocompatible advantages of green-synthesized nanoparticles from various biological sources encourage us to evaluate the anticancer benefits of MgONPs synthesized from pumpkin seeds using clean, green, inexpensive, ecofriendly, reliable, and safe approach.

Herein, we evaluate the anticancer effect of green-synthesized MgONPs against ovarian teratocarcinoma cell line (PA-1) using various *in vitro* tests. The current study is expected to provide a realistic *in vitro* tool against ovarian carcinoma that enables primary anticancer screening of MgONPs, and preventing the entry of drugs with insufficient antitumor activity from entering preclinical animal testing. The significant challenge of our study could be a poor correlation between preclinical *in vitro* and *in vivo* data with clinical trials. However, due to the proper screening of anticancer agents, identifying the most effective *in vitro* cancer model is expected to reduce the financial burden and time at later stages.

## Materials and methods

All reagents and solvents used in this study were procured from commercial sources (Merck & Sigma, India) without further purification. Streptomycin, penicillin, magnesium nitrate, fetal bovine serum (FBS), nitric acid, Dulbecco’s Modified Eagle Medium (DMEM), phosphate-buffered saline (PBS), 3-(4,5 dimethylthiozol-2-yl)-2,5-diphenyl tetrazolium bromide (MTT), 2′7 ‘diacetyl dichlorofluorescein (DCFH), trypsin-EDTA, acridine orange, and ethidium bromide were obtained from these companies. All other chemicals were purchased locally and were of analytical grade.

### Preparation of the extract

A fresh pumpkin (*C. maxima*) was procured from Jagatpura, Jaipur, India. As per the institutional ethical approval committee, no formal approval was required to conduct this study. The specimen was confirmed by Dr. A. Khan (Department of Botany, AMU, Aligarh, India) and was deposited in a departmental herbarium containing voucher number 84/21. 5.0 g pumpkin seeds were dried and grinded to a fine powder. The powdered seeds were refluxed for 1 h in deionized water in a round-bottom flask and cooled at room temperature (RT). The resultant solution was filtered through Whatman filter paper to obtain a purified crude extract. The filtrate was then stored in a cool environment for future requirements.

### Biosynthesis of magnesium oxide nanoparticles from pumpkin seed extracts

**FIGURE 1:**
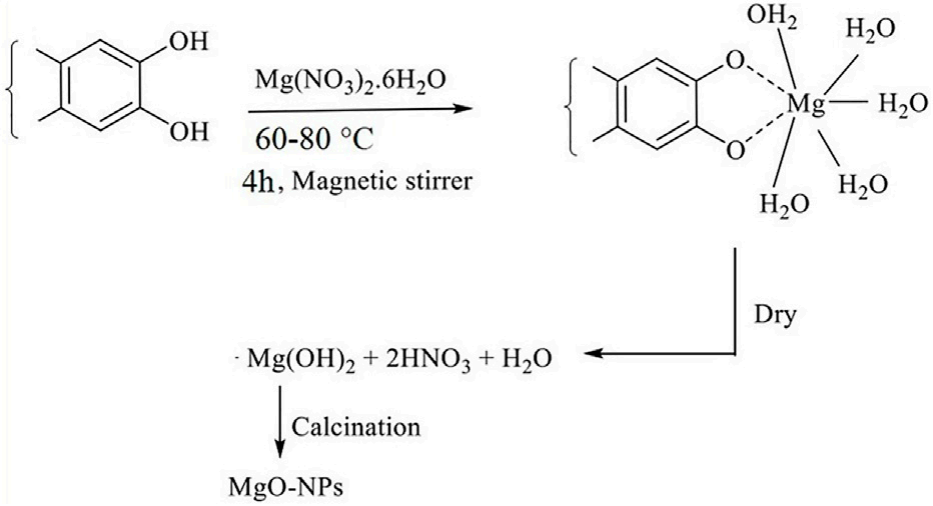
Tentative mechanism of MgONPs synthesis from pumpkin seed extracts.

The biogenic synthesis of MgONPs required 20 ml of pumpkin seed extracts and 80 ml of an aqueous solution of Mg(NO_3_)_2_ (0.1 M) under continuous magnetic stirring for 4 h at 60–80°C. As a result, the solution developed a brownish colloidal appearance, indicating the formation of MgONPs. The solution was then centrifuged at 10,000 rpm for 10 min, dried in an oven at 50–60°C, and calcinated in a muffle furnace at 500°C to get the white powder of MgONPs. A tentative mechanism of MgONP biosynthesis is depicted in Figure 1.

### Characterization of biogenic magnesium oxide nanoparticles

The formation of MgONPs was confirmed by UV–vis absorption spectroscopy (UV-1800, Japan) with a resolution of 1 nm ranging from 200–800 nm. Moreover, Fourier-transform infrared spectroscopy (FTIR) analysis was applied to detect and measure the functional group of pumpkin seed extracts involved in synthesizing MgONPs in the range of 4,000–400 cm^−1^ (Perkin Elmer Spectrum 2000 FTIR) by the KBr pellet technique. Cu Kα radiation was employed to determine the X-ray diffraction (XRD) spectrum of MgONPs at 40 kV. The obtained XRD pattern of synthesized nanoparticles was compared with the Joint Committee on Powder Diffraction Standards (JCPDS) file. The characteristic diffraction peak in the XRD pattern of MgONPs was indexed as the face-centered cubic phase. In addition, the particle distribution, elemental composition, and surface morphology of biosynthesized MgONPs were evaluated by scanning electron microscopy (Nova nano, FE-SEM 450 FEI). The particle size and shape were confirmed by transmission electron microscopy (TEM, TECNAI G-20).

### Cell line and culture medium

The ovarian teratocarcinoma cell line (PA-1) was procured from the National Centre for Cell Science (NCCS), Pune, India. The cells were grown in DMEM high-glucose media with 100 units/ml of penicillin/streptomycin and 10% fetal bovine serum and maintained in a humidified atmosphere of 5% CO_2_ at 37°C.

### Cytotoxicity assay (MTT)

The cytotoxicity of MgONPs was evaluated by 3-(4,5-dimethylthiazol-2-yl)- 2,5-diphenyltetrazolium bromide (MTT) dye reduction assay, which is dependent on the conversion to the water-insoluble blue formazan compound by mitochondrial dehydrogenases (Alharthy et al., 2022). In brief, PA-1 cells were harvested and seeded in a 96-well plate and exposed to different concentrations (2.5–17.5 μg/ml) of MgONPs for 24 h at 37°C in 5% CO_2_. After the treatment, the medium was aspirated, and the treated cells were exposed to 10 µl of MTT for another 2 h in the dark at 37°C. At the end of the incubation period, the purple formazan crystals were solubilized with 50 µl DMSO and measured spectrophotometrically on a microplate reader at 595 nm. The following formula calculated the percentage of cytotoxicity:

$$\text{Cell}\;\text{proliferation}\;\text{Inhibition}\;\left(\%\right)=\frac{\text{Mean}\;\text{absorbance}\;\text{of}\;\text{the}\;\text{control}\;\text{cells}-\text{Mean}\;\text{absorbance}\;\text{of}\;\text{the}\;\text{treated}\;\text{cells}\;}{\text{Mean}\;\text{absorbance}\;\text{of}\;\text{the}\;\text{control}\;\text{cells}}\;X\;100.$$

The concentrations of MgONPs showing 50% reduction in cell viability (IC_50_) were evaluated by extrapolating the dose–response curve.

### Morphological alteration of PA-1 cells by light microscopy

After treating with different concentrations of MgONPs, the PA-1 cells were examined under a light microscope (×10 magnification). The photomicrographs of the cells were thoroughly evaluated for morphological changes such as shrinkage, detachment, membrane blebbing, and distorted shape.

### Analysis of apoptotic induction in PA-1 cells

To investigate apoptosis or necrosis, acridine orange/ethidium bromide (AO/EB) double fluorescent labeling was used according to the method described by Tabrez et al. (2022b). Briefly, the cells were seeded in a 6-well plate (5 × 10^4^ cells/well) and treated with different concentrations of MgONPs (10, 12.5, and 15 μg/ml) for 24 h. After treatment, the cells were washed with cold PBS and stained with a mixture of AO/EB (1:1; 100 μg/ml) for 5 min. After staining, the cells were monitored by using a fluorescence microscope (×40 magnification). The number of cells showing the feature of apoptosis was counted as a function of the total number of cells.

### Investigation of reactive oxygen species production in PA-1 cells

The intracellular reactive oxygen species (ROS) level was measured by the dichloro-dihydro-fluorescein diacetate (DCFH-DA) assay, as mentioned previously (Alharthy et al., 2022; Tabrez et al., 2022b). The PA-1 cells were seeded in 6-well plates (2 × 10^6^ cells/well) and treated with different concentrations of MgONPs (10, 12.5, and 15 μg/ml) for 24 h at 37°C. After treatment, the cells were washed with PBS, and 25 μM of DCFH-DA was added for 30 min at 37°C. After that, the cells were washed with DMEM, and the fluorescence was recorded every 5 min, over to 30 min (excitation 485 nm, emission 535 nm), using a spectrofluorometer (Shimadzu, Columbia, United States). The increase in ROS production was calculated by a mean slope/min and normalized to the unexposed control.

### Cell adhesion assay

The cells were separated at 0, 15, 30, 45, and 60 min intervals in 6-well plates, and the wells were rinsed with PBS to remove the weakly attached/unattached cells. The seeded cells were fixed with paraformaldehyde (5%) and crystal violet dye (1%) and fostered for 15 min. After incubation, crystal violet dye was bound to cellular proteins, and excess crystal violet was washed with PBS. The amount of crystal violet destained to protein was proportional to the number of cells in the well.

### Wound healing (scratch) assay

The scratch assay was performed according to the protocol defined by Goel and Gude (2011). In brief, the PA-1 cells were plated to make an 80% confluent monolayer. The monolayer of cells was scratched by using a sterile 200-microliter pipette tip to make a straight line to create scratches. After cleansing with PBS, the cells were treated with different doses of MgONP (10, 12.5, and 15 μg/ml) and maintained at 5% CO_2_ and 37°C for 36 h. Later, the wound closure level was assessed, and microphotographs were taken at 0 and 24 h.

### Statistical analysis

Data are presented as the mean ± SD of three independent values (wherever applicable). The control and treated cells were compared using a one-way ANOVA. *p* < 0.05 was considered as statistically significant.

## Results and discussion

Different spectroscopic techniques confirmed the biosynthesis of MgONPs from the pumpkin seed extract. The bioactive moieties in pumpkin seed extracts act as bioreducing, stabilizing, and capping agents and enhance the potential of biogenic MgONPs.

### UV–visible spectrophotometry spectroscopy confirmed the synthesis of biogenic magnesium oxide nanoparticles and their stability

**FIGURE 2:**
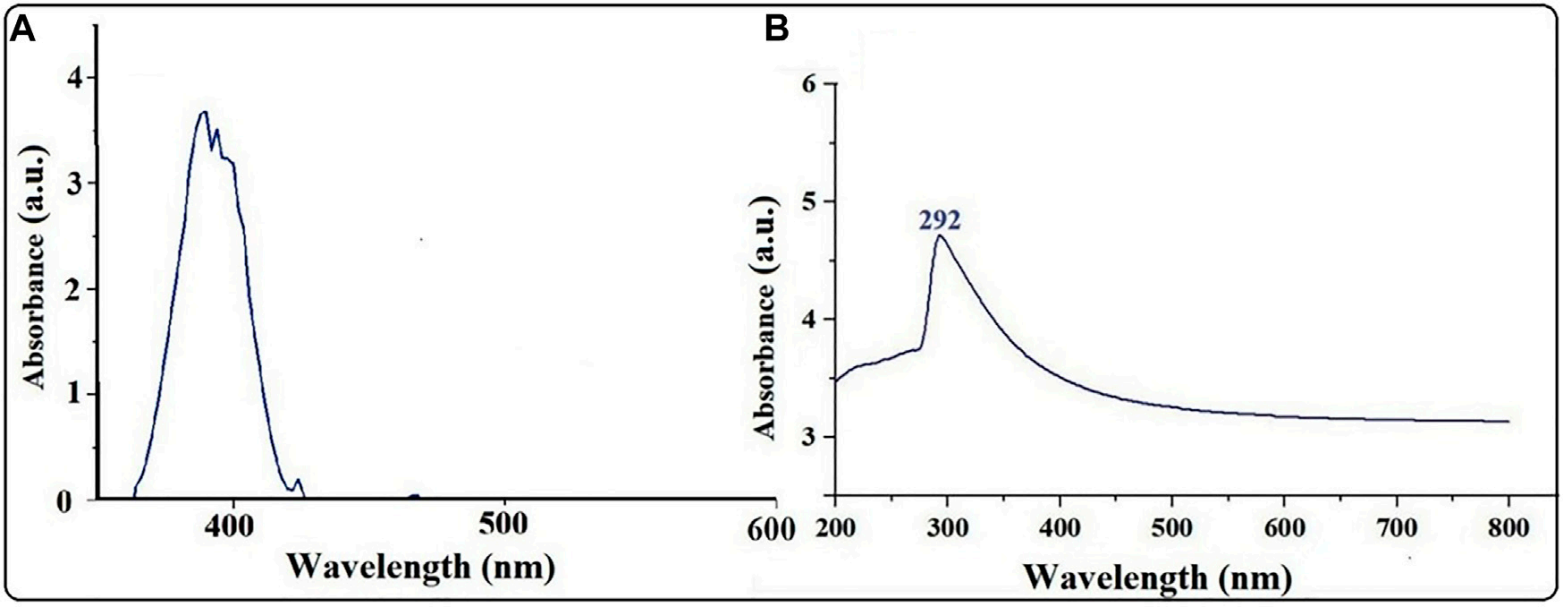
**(A)** UV–vis spectra of pumpkin extract. **(B)** UV–vis spectra of MgONPs synthesized from pumpkin seed extracts.

The UV–vis spectrum of the biosynthesized MgONPs showed a peak at 292 nm, confirming the formation of small-sized nanoparticles (Figure 2). Single-surface plasmon resonance (SPR) bands that form at short wavelengths below 300 nm in UV–visible spectroscopy indicate the presence of small-sized particles. In contrast, other absorption peaks indicated the existence of several bioactive compounds, which may be essential for Mg(NO_3_)_2_ reduction to MgONPs (Pugazhendhi et al., 2019). Various studies have reported the UV–vis absorption spectra of MgONPs in the range of 250–350 nm synthesized from different biological sources (Pugazhendhi et al., 2019; Amina et al., 2020; Fouda et al., 2022). In our study, MgONPs were extracellularly synthesized and could be easily purified (Fouda et al., 2022). The pumpkin seeds contained large amounts of β- and γ-tocopherol, which are beneficial as reducing and capping agents that help in the synthesis of NPs (Shankar and Rhim, 2016; Kulczyński and Gramza-Michałowska, 2019). We used nitric acid during NP synthesis, which helps to synthesize NPs of smaller size, lower magnetization, better thermal properties, and higher stability (Nurdin et al., 2014).

### Fourier-transform infrared spectroscopy analysis

**FIGURE 3:**
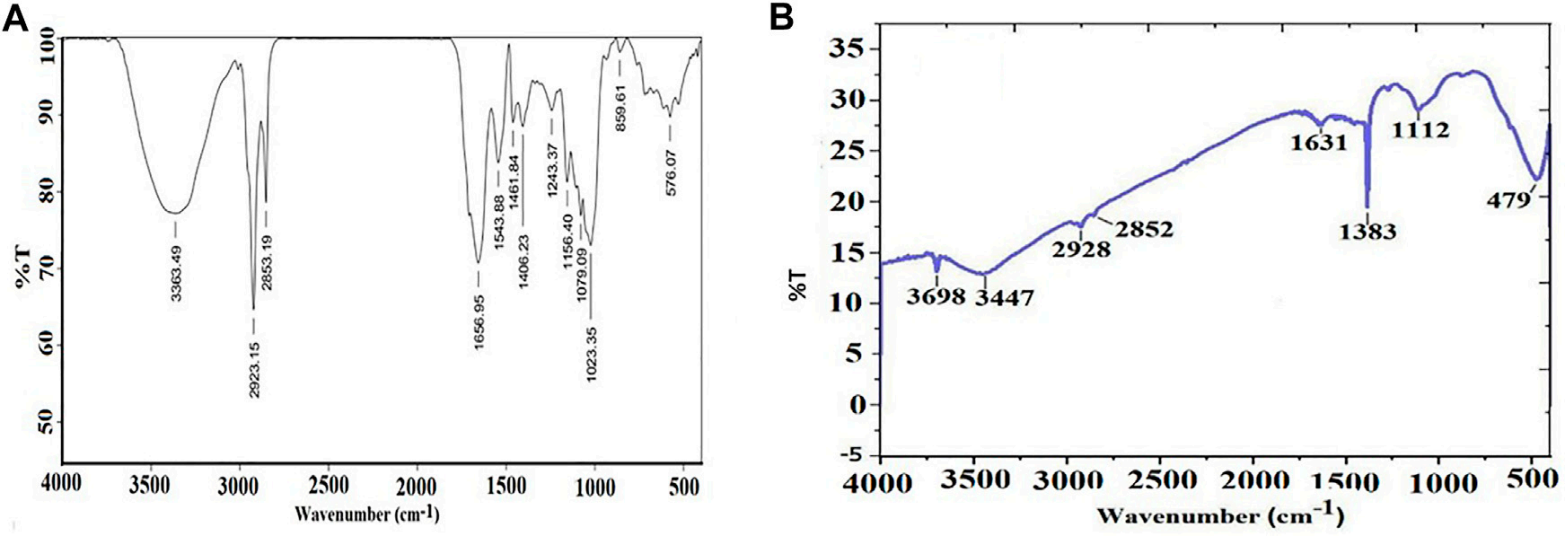
**(A)** FTIR spectra of pumpkin seed extracts. **(B)** FTIR spectra of MgONPs.

FTIR spectra of the pumpkin seed extracts (Figure 3A) and biosynthesized MgONPs (Figure 3B) were monitored at the wavelength range of 4,000–400 cm^−1^. The major IR vibration functional bands in the MgONPs spectrum were recorded at 479 cm^−1^ (Figure 3B). The different bioactive moieties in pumpkin seed extracts act as reducing agents in synthesizing stable nanoparticles. The IR bands that appeared previously could be of an ester, ethers, carbonyl (polyols), or aromatic compounds. On the other hand, the peaks observed at 3,696 and 3,447 cm^−1^ can be assigned to the hydrogen-bonded hydroxyl and free NH_2_ groups. Similarly, the peaks around 2,928, 2,852, 1,631, 1,343, and 1,112 cm^−1^ are attributed to the stretching vibration of the aromatic C=C bond, while the peak at 479 cm^−1^ indicates the formation of MgONPs (Sharma et al., 2020). The FTIR analysis confirmed the role of bioactive compounds as reducing, capping, and stabilizing agents in MgONP biosynthesis. Our FTIR spectra are similar and concomitant with previously reported studies (Pugazhendhi et al., 2019; Amina et al., 2020; Fouda et al., 2022).

### X-ray diffraction analysis

**FIGURE 4:**
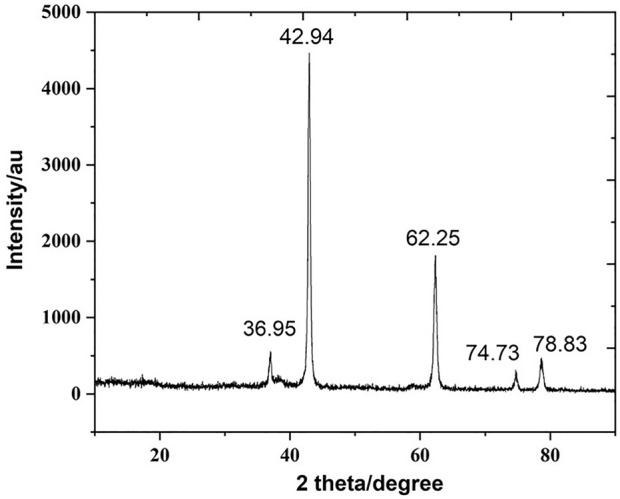
XRD pattern of biogenic MgONPs.

The XRD pattern of biogenic MgONPs was verified by the characteristic peak in the XRD image (Figure 4). The diffraction peaks at 2 theta values of 36.95°, 42.94°, 62.25°, 74.73°, and 78.83° are identified in the (111), (220), (220), (311), and 222) planes, respectively, of a faced center cubic lattice of magnesium, indicating the crystalline nature of MgONPs confirming the earlier study (Safaei-Ghomi et al., 2015). Moreover, a standard diffraction pattern of MgONPs (JCPDS file number 89-4248) showed similar patterns in the XRD of nanoparticles.

### Scanning electron microscopy analysis

**FIGURE 5:**
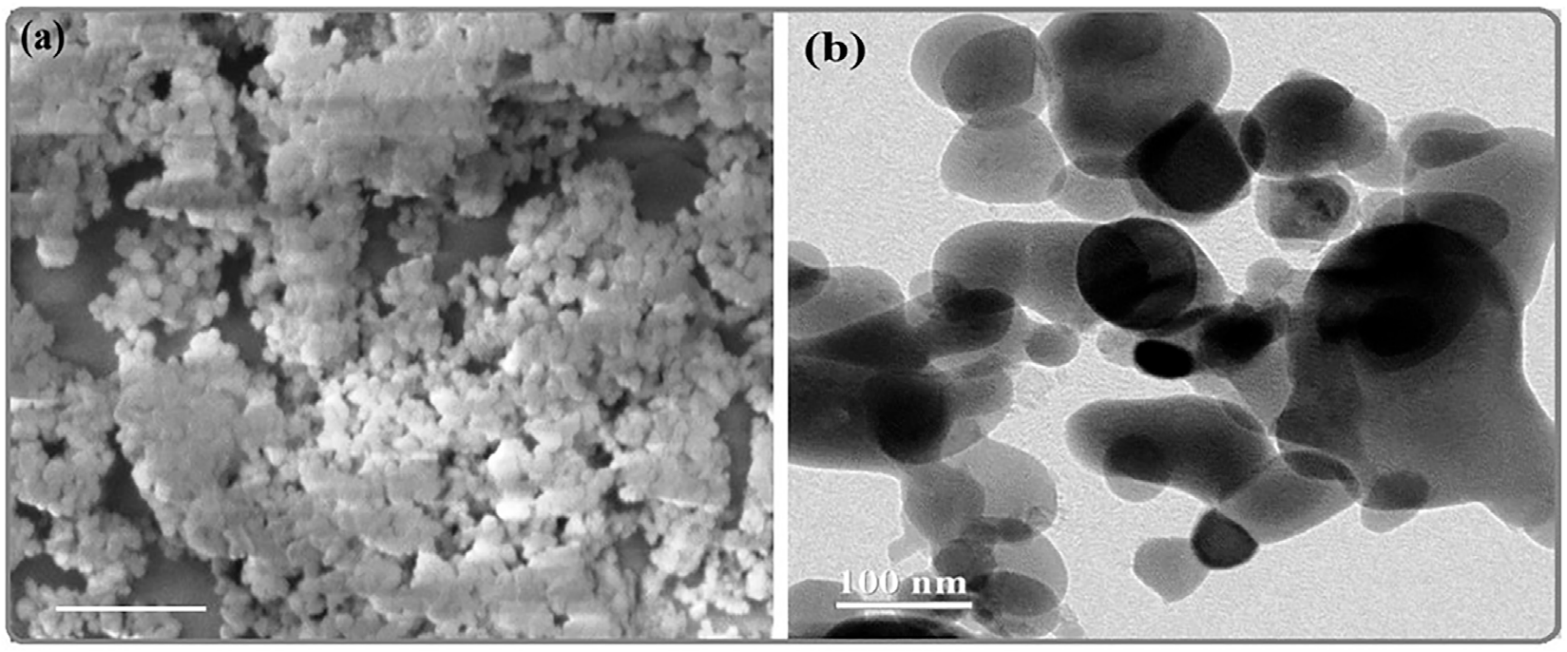
**(A)** SEM image of MgONPs. **(B)** TEM image of MgONPs.

The morphology of biosynthesized MgONPs was characterized by SEM analysis at different magnifications. It showed agglomeration, resulting in moderately dispersed and slightly coalesced MgONPs (Figure 5A). The MgONPs were found to be of different sizes (<100 nm) and distinguished shapes. The observation of biosynthesized nanoparticles was cluster and randomly distributed forms. Similar data have been reported in the previous studies related to MgONPs biosynthesized from different sources (Pugazhendhi et al., 2019; Kgosiemang et al., 2020; Pamidimukkala, 2021).

### Transmission electron microscopy analysis

The potential of nanoparticles is usually correlated with different characters that include shape, size, and distribution (Fouda et al., 2022). The electron diffraction patterns by TEM analysis identified the phase of nanoparticles and determined their particle size. The TEM image showed that the particles are nearly spherical and eclipsed in morphology (Figure 5B). The nanoparticles were moderately dispersed, and the particles’ average size was ∼100 nm. Earlier reports have also observed similar sizes of biogenic MgONPs synthesized from various sources (Amina et al., 2020; Fouda et al., 2022). Given the earlier scientific reports, the current MgONPs sized <100 nm are expected to be used in various biomedical and biotechnological applications.

### Energy dispersive X-ray analysis (EDX)

**FIGURE 6:**
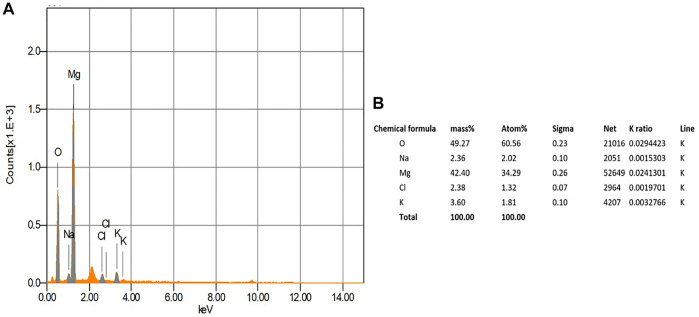
EDX analysis of the biogenic Mg0NPs.

The EDX analysis is used to detect the elemental composition of the biosynthesized MgONPs (Fouda et al., 2022). The production and quality of the synthesized nanomaterials are related to the existence of significant peaks in the EDX spectrum. Figure 6A shows an EDX spectrum of biogenic MgONPs that include atoms and mass percentages with a distinct peak corresponding to Mg and O and peaks of Na, Cl, and K in small amounts from sample preparation. Magnesium showed a high peak at approximately 1.2–1.3 keV, while oxygen showed a peak at approximately 0.5 keV. The quantitative analysis of the sample revealed the mass% of Mg and O ions was 42.4 and 49.3%, respectively, whereas the atomic percentages were 34.3 and 60.6%, respectively (Figure 6B). The compositions of the other elements were negligible, indicating the highly pure nature of our biogenic MgONPs. The EDX profile of MgONPs synthesized from algal extract also showed the weight percentages of Mg and O ions as 54.1 and 20.6%, respectively (Fouda et al., 2022). Another study also showed the average elemental percentage of Mg and O as 38.2%, and 27.9% in MgONPs biosynthesized from *S. costus* (Amina et al., 2020).

### Cytotoxicity and morphological alterations

**FIGURE 7:**
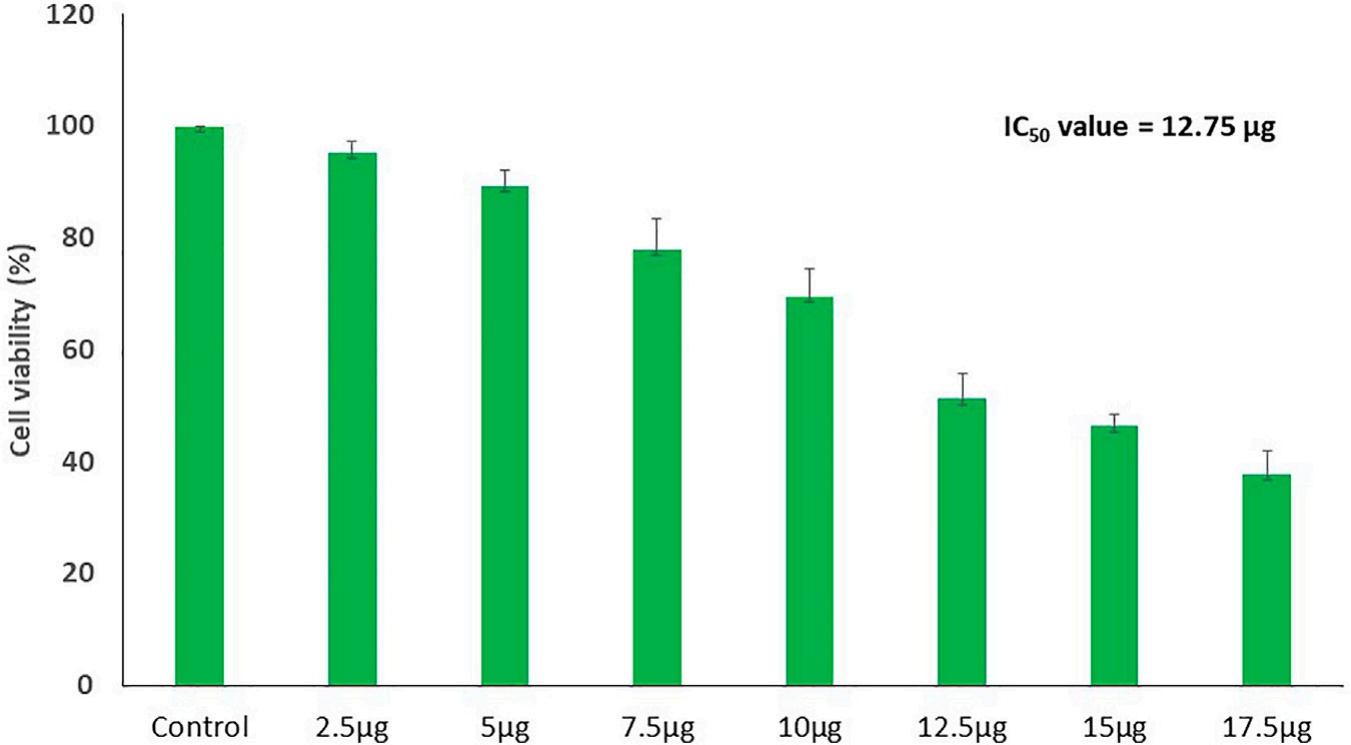
Dose-dependent decline in the cell viability of PA-1 cells after treating with variable concentrations of MgONPs.

The results showed a concentration-dependent decrease in cell viability in PA-1 cells compared to the untreated control (Figure 7). The viability of cells starts declining at the lowest tested concentration of 2.5 μg/ml, and at the highest concentration (17.5 μg/ml), it was found to be 37.7% only. The IC_50_ concentrations of biosynthesized MgONPs were found to be 12.5 μg/ml. Several researchers have also biosynthesized MgONPs from different sources, but the recorded IC_50_ dose was much higher, indicating better anticancer efficacy of our biosynthesized MgONPs (Pugazhendhi et al., 2019; Amina et al., 2020; Fouda et al., 2022). The cytotoxicity could also depend on the types of cancer cells having abnormal metabolism and morphology, size, and shape of NPs, which in turn varies with the preparation method (Perde-Schrepler et al., 2019). Based on the estimated IC_50_ value, three different concentrations (10, 12.5, and 15 μg/ml) of MgONPs were selected for the subsequent experiments.

**FIGURE 8:**
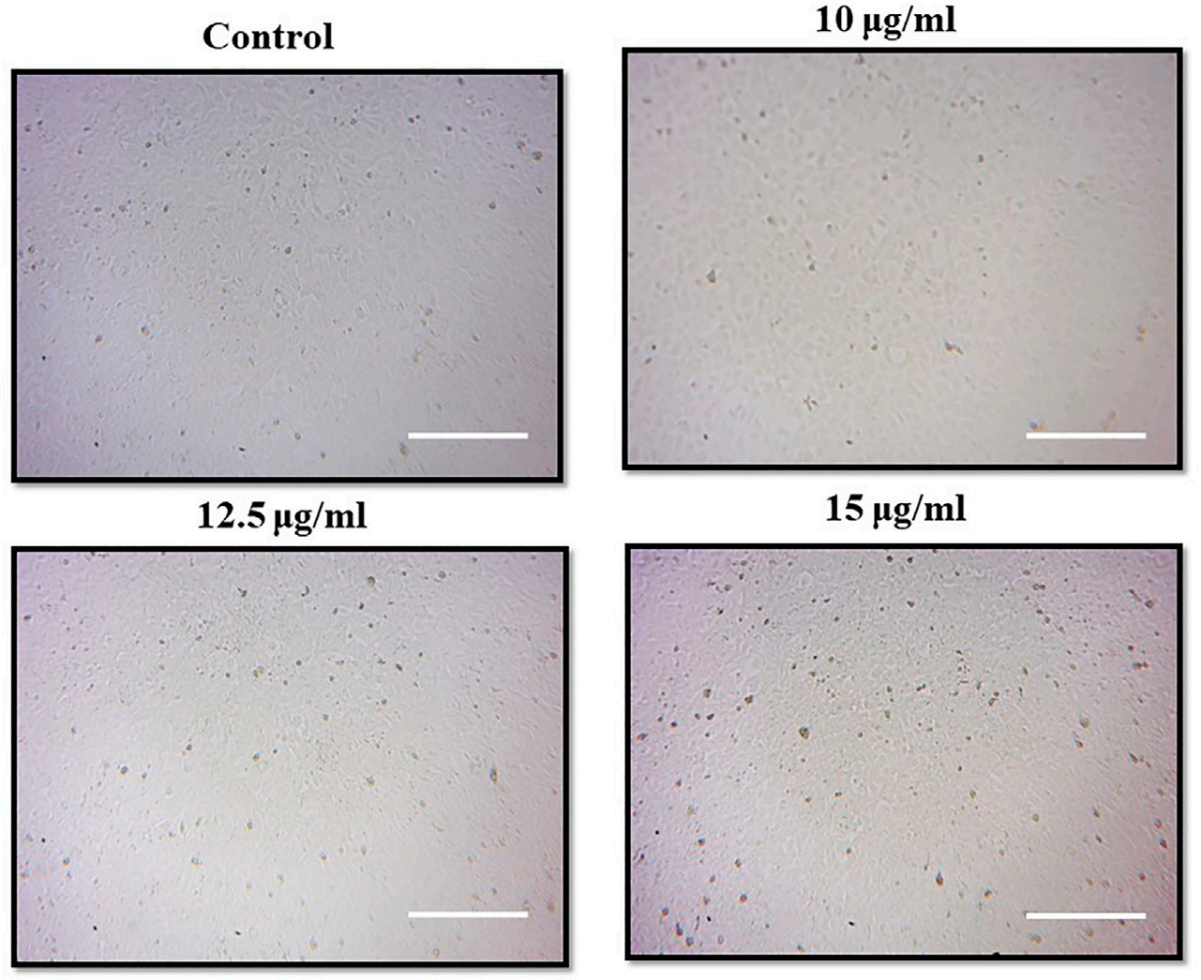
Morphological changes in PA-1 cells after the treatment with MgONPs.

The photomicrographs of MgONPs-treated PA-1 cancer cells showed a loss/alteration in cell morphology in a concentration-dependent manner (Figure 8). Cell shrinkage, detachment, membrane blebbing, and distorted shape were observed at the highest concentration. However, the cell morphology of the untreated control cells was found to be normal and intact. The entry of MgONPs into mammalian cells occurs mainly through endocytosis or macropinocytosis (Fouda et al., 2022). A similar alteration in cell morphology was reported earlier by green-synthesized MgONPs in different cancer cell lines, implying that the cytotoxicity of synthesized MgONPs may be attributed to their antineoplastic characteristics and ability to trigger cell death *via* a variety of molecular mechanisms (Farah et al., 2016; Al-Sheddi et al., 2018). Pugazhendhi et al. (2019) observed a similar pattern of cell rounding and shrinkage, membrane blebbing, apoptotic body formation, chromatin condensation, and reduced cell population after the treatment with MgONPs in A549 cells.

### Induction of apoptosis by magnesium oxide nanoparticle treatment

**FIGURE 9:**
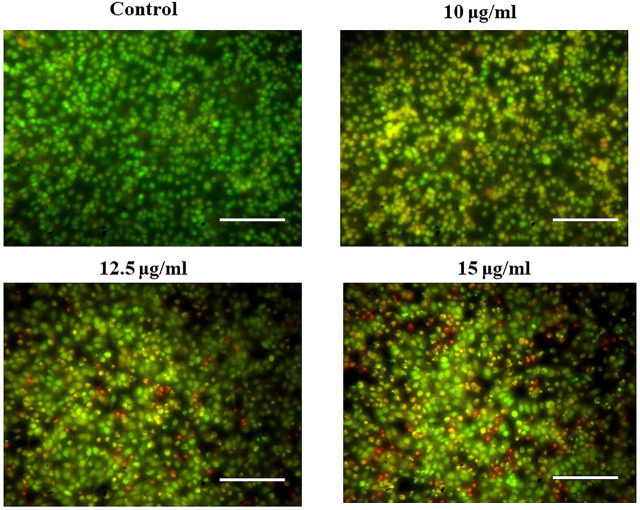
Treatment of MgONPs resulted in induction of apoptosis.

Figure 9 illustrates the AO/EB fluorescence pattern of PA-1 cells treated with MgONPs and showed the presence of early and late apoptotic cells, suggesting the apoptotic potential of MgONPs. Most of the untreated control cells showed an intact morphology and normal nuclear membrane. At 10 μg/ml MgONPs treatment, the PA-1 cells showed the initiation of nuclear fragmentation. However, MgONP treatment at higher concentrations (12.5 and 15 μg/ml) showed more intense nuclear fragmentation and advanced apoptotic cells.

During double-dye (AO/EB) staining, the AO permeates all the cells and give green fluorescence. On the other hand, EB interacts with the DNA of those cells that have lost their membrane integrity and stains the nuclei red for apoptotic cells. Double staining is a convenient, economical, and reliable *in vitro* tool to distinguish normal cells, early apoptotic cells, late apoptotic cells, dead cells, and nuclear morphology (Liu et al., 2015). The AO/EB staining interprets live cells as the usual green nucleus, early apoptotic cells as yellow fluorescence, which indicates a condensed or fragmented nucleus containing chromatin, and late apoptotic cells as orange fluorescence, which indicates chromatin condensation or fragmentation and cell necrosis (Liu et al., 2015). Dose-dependent cell growth inhibitions could happen due to the microscopically visible cell permeability disruption, reactive oxygen species (ROS) production, activation of caspase cascade-mediated apoptosis, and cell cycle arrest (Martínez-Torres et al., 2018). Moreover, the apoptotic induction and associated DNA damage have also been linked to excessive production of ROS, oxidative stress, and Sub-G1 arrest of cancer cells (Salehi et al., 2018). The modulation of apoptotic pathways is challenging as several pathways are involved in this process. However, our biosynthesized MgONPs showed promising apoptosis-inducing potential against ovarian cancer cells. Our results agree with the earlier reports that have observed apoptosis induction *via* different mechanisms by MgONPs, green synthesized from various sources against different cancer cell lines (Pugazhendhi et al., 2019; Amina et al., 2020; Fouda et al., 2022).

### Magnesium oxide nanoparticles induce intracellular reactive oxygen species generation

**FIGURE 10:**
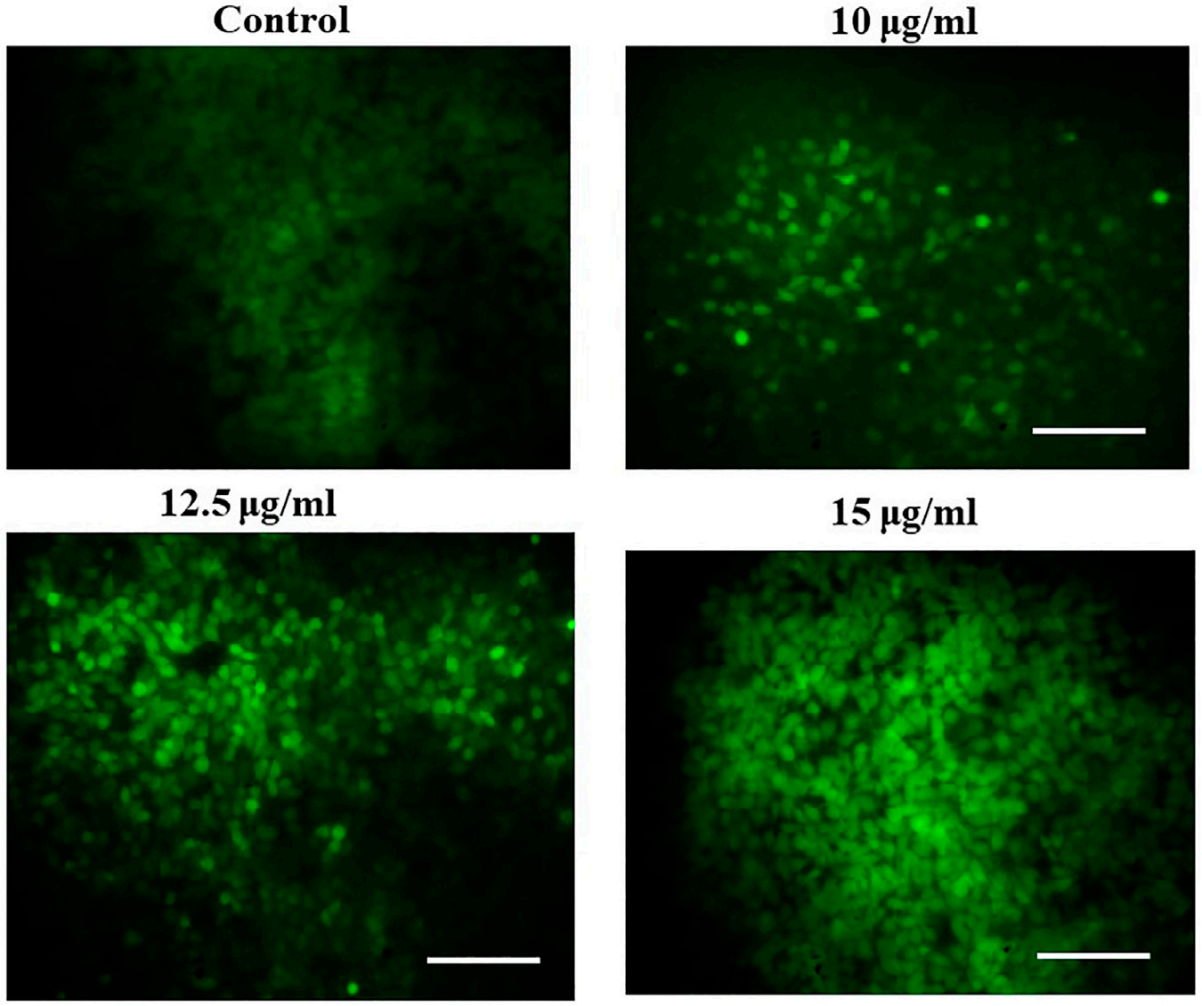
Effect of MgONPs on intracellular ROS generation in PA-1 cells.

We observed a dose-dependent rise in ROS production in MgONP-treated PA-1 cells (Figure 10). The untreated control cells showed a dull green fluorescence. However, the MgONPs-treated (10, 12.5, and 15 μg/ml) cells showed a strong DCF stained green fluorescence indicating the production of intracellular ROS. The intricate association of ROS levels and cancer progression depends on the precise control of ROS generation and their scavenging. Cancer cells have more evolved antioxidant systems than normal cells and survive at slightly higher ROS levels than their normal counterparts. This characteristic makes cancer cells more vulnerable to outside factors, upregulating the generation of ROS. Increasing the ROS levels explore a growing number of therapeutic approaches to exceed the redox adaption of the same cells (Perillo et al., 2020). Our result corroborated well with the earlier studies that report a rise in ROS production in response to MgONPs treatment (Behzadi et al., 2018; Pugazhendhi et al., 2019). Various nanoparticles have been reported to induce ROS formation, eventually stimulating various anticancer events, such as oxidative stress, lipid peroxidation, DNA damage, and destabilization of mitochondria, resulting in cell death by apoptosis and necrosis (Alharthy et al., 2022; Tabrez et al., 2022b; Zughaibi et al., 2022). A low level of ROS has been suggested to regulate cancer cell tumorigeneses, whereas a high-level causes severe cellular damage (Youhannayee et al., 2019; Silva et al., 2021). Some studies also indicated that treating cells with various stress factors, such as anticancer drugs, increases ROS levels and induces cellular apoptosis by triggering proapoptotic signaling molecules (Lampiasi et al., 2009; Azizi et al., 2017). It is also assumed that oxidative stress-causing factors can selectively lead to cancer cell death compared with normal cell due to the higher production of ROS (Azizi et al., 2017).

### Effect of magnesium oxide nanoparticles on the cell adhesion of PA-1 cells

**FIGURE 11:**
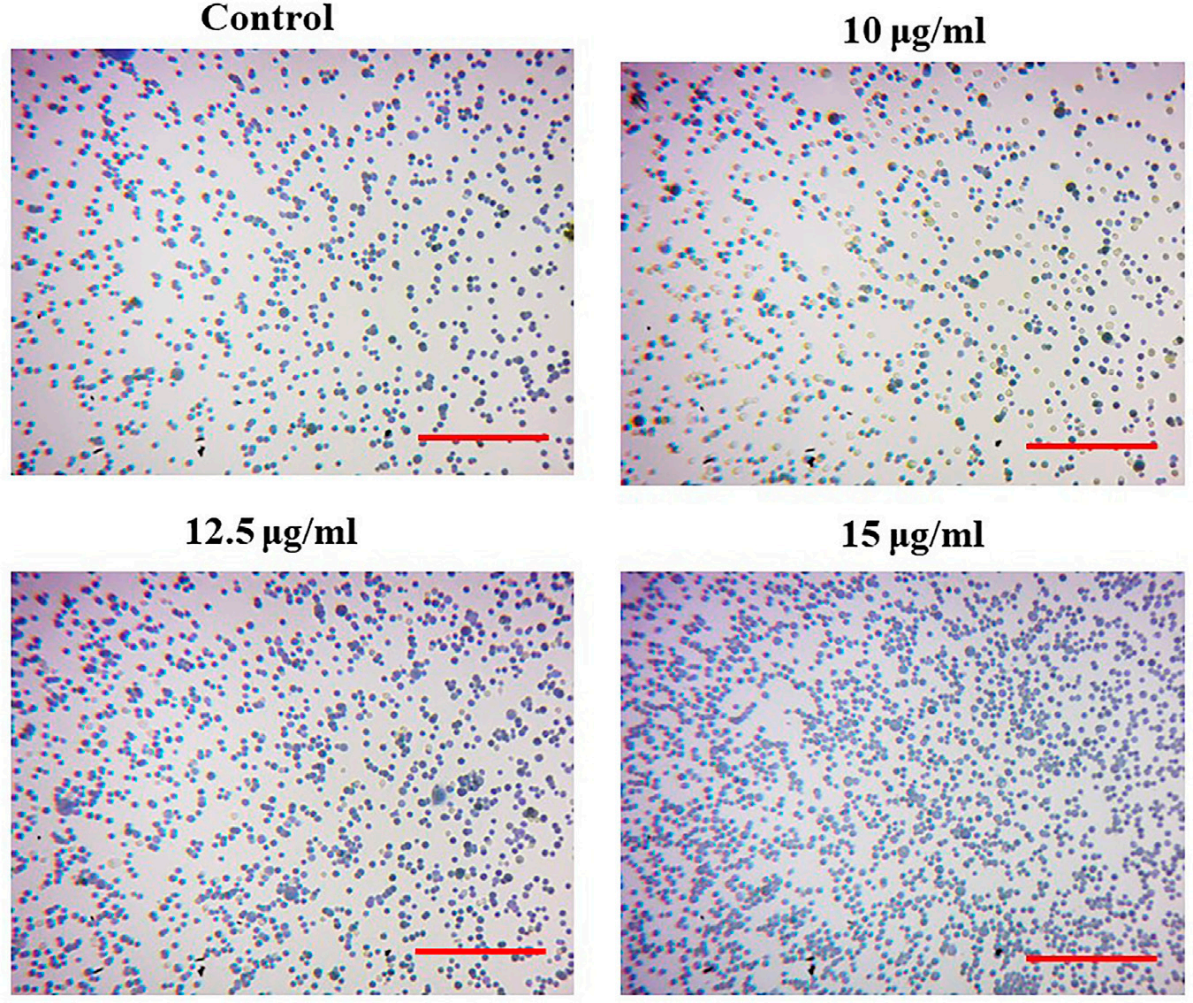
Effects of MgONPs on cell adhesion in PA-1 cells.

The cell adhesion assay evaluates the ability of cell attachment to the extracellular matrix. In this assay, the PA-1 cells were implanted with various doses of MgONPs (10, 12.5, and 15 μg/ml). The MgONP-treated cells showed an aberrated cell adhesion pattern (Figure 11). These abnormal cells could reduce cancer progression and metastasis (Lin et al., 2020; Pallavi et al., 2020).

### Cell migration inhibition by wound scratch assay in PA-1 cells

The cell migration assay is an essential aspect of cancer cell progression, which utilizes the ability of cells to migrate. The wound scratch assay showed a significant wound closure in untreated control PA-1 cells. However, the MgONP-treated cells showed a delay in the closure of the scratched area, suggesting a decrease in the motility and migration of cancer cells. The remnant scar region was found to be the highest compared to the starting gap area at the highest tested concentration of MgONPs (15 μg/ml). Our results showed a similar trend in inhibiting PA-1 cell migration as observed by other anticancer agents (Das et al., 2020; Haque et al., 2021). For comparison purposes, we have enlisted a table comprising the efficacy of green-synthesized MgONPs from various sources (Table 1). As far as our knowledge is goes from the available scientific literature, we are reporting the anticancer potential of green-synthesized MgONPs from pumpkin seed extracts for the first time. Experimental evidence suggests fascinating nutraceutical qualities of pumpkin seeds because they possess many bioactive compounds (Dotto and Chacha, 2020). Pumpkin seeds are a rich source of health-promoting compounds such as fatty acids, tocopherols, carotenoids, phenolic compounds, phytosterols, squalene, and minerals (Gedi and Mariod, 2022). A recent report suggests an inverse association of the risk of several types of cancer, such as breast, rectal, and lung cancer, to pumpkin seed intake (Batool et al., 2022).

**TABLE 1:** Comparative efficacy of green-synthesized MgONPs from various sources.

Biological source	Cancer model	Anticancer effects	IC_50_/treatment time	References
*Cystoseira crinita*	Caco-2 colon cancer cells	*In vitro* cytotoxicity	113.4 μg/ml/48 h	Pugazhendhi et al. (2019)
*Allamanda cathartica L*	hPBMC cells	*In vitro* cytotoxicity	∼55 μg/ml/72 h	Nurdin et al. (2014)
*Saussurea costus (Qustal Bahri)*	MCF-7 breast cancer cells	*In vitro* cytotoxicity, release in LDH, morphological alterations, depolarization of mitochondrial membrane potential, increase in intracellular ROS production	60 μg/ml/48 h	Amina et al. (2020)
*Euphorbia tirucalli*	MCF-7 breast cancer cells	*In vitro* cytotoxicity	10 μg/ml/48 h	Shankar and Rhim (2016)
*Calotropis gigantea*	Embryonic zebrafish	*In vivo molecular* toxicity, increase in intracellular ROS production, induction of apoptosis	520 μg/ml	Das et al. (2020)
*Sargassum wightii*	A-549 lung cancer cells	*In vitro* cytotoxicity	37.5 μg/ml/24 h	Fouda et al. (2022)
*Penicillium* sp.	A-549 lung cancer cells	*In vitro* cytotoxicity, induction of apoptosis	100 μg/ml/24 h	Haque et al. (2021)
*Costus pictus D. Don*	Dalton’s ascites (DLA) cells	Inhibition in cellular migration and reduced cell viability	200 μg/ml/3 h	Dotto and Chacha (2020)
*Lactobacillus* sp.	HL-60 cancer cells	HL-60 cancer cell lines	100 μg/ml/24 h	Gedi and Mariod (2022)

Overall, the cellular modifications we observed could be explained as the invaded MgONPs induced ROS production, damaging the mitochondrial membrane integrity and activating the apoptotic pathway, resulting in cell death (Fouda et al., 2022). Subsequently, MgONPs’ potential to cause cellular injury could also be size-dependent, where smaller MgONPs increased ROS generation, improved interactions with cellular components, and improved membrane penetration to release Mg^+^ ions (Fouda et al., 2022). Despite their therapeutic potential, the challenge is appropriate bioavailability and cancer targeting by the compounds present in pumpkin seeds. The nanoparticle-based formulations are expected to overcome these challenges, and could provide substantial benefits. Even though the exact mechanism of MgONPs action is still obscure, it has been reported to induce apoptosis and ROS formation. Our results are concurrent with earlier reports, highlighting the significant therapeutic potential of our biogenic MgONPs against the studied cell lines.

## Conclusion

The biogenically synthesized MgONPs seem to be a promising anticancer agent, considering their wide range of medicinal applications. This nanoformulation showed considerable cytotoxicity, apoptotic induction, increased ROS formation, inhibition, and migration of ovarian teratocarcinoma cell line, highlighting its anticancer potential. However, additional work, especially in different *in vitro* and *in vivo* models, is recommended to find out their real potential before this environment-friendly and cost-effective nanoformulation could be exploited for the benefit of humankind.

## Data Availability

The original contributions presented in the study are included in the article/Supplementary Material. Further inquiries can be directed to the corresponding authors.
